# Anti-Aging Properties of Chitosan-Based Hydrogels Rich in Bilberry Fruit Extract

**DOI:** 10.3390/antiox13010105

**Published:** 2024-01-15

**Authors:** Elżbieta Studzińska-Sroka, Magdalena Paczkowska-Walendowska, Cansu Erdem, Jarosław Paluszczak, Robert Kleszcz, Marta Hoszman-Kulisz, Judyta Cielecka-Piontek

**Affiliations:** 1 Department of Pharmacognosy and Biomaterials, Poznan University of Medical Sciences, Rokietnicka 3 Str, 60-806 Poznań, Poland; elastudzinska@ump.edu.pl (E.S.-S.); martahoszman@wp.pl (M.H.-K.); jpiontek@ump.edu.pl (J.C.-P.); 2 Department Pharmaceutical Chemistry, Ege Üniversitesi, 35040 İzmir, Turkey; cansueerdem@gmail.com; 3 Department of Pharmaceutical Biochemistry, Poznan University of Medical Sciences, Rokietnicka 3 Str, 60-806 Poznań, Poland; paluszcz@ump.edu.pl (J.P.); kleszcz@ump.edu.pl (R.K.)

**Keywords:** bilberry fruits, blueberry fruit, anti-aging potential, topical preparation, cosmetics, chitosan

## Abstract

Photoaging is a process related to an increased level of reactive oxygen species (ROS). Polyphenols can scavenge free radicals in the body, which can delay skin aging. Therefore, our work aimed to prepare a biologically active extract from dry fruits of *Vaccinium myrtillus* or *Vaccinium corymbosum* and use it for the preparation of hydrogels for topical application. Therefore, eight different extracts (using *V. myrtillus* and *V. corymbosum* and different extraction mixtures: methanol, methanol–water 1:1, water, acetone–water 1:1) were prepared and their phytochemical (total polyphenolic content, total flavonoid content, total anthocyanin content) and biological properties (antioxidant, anti-hyaluronidase, and anti-tyrosinase activity) were assessed. Cytotoxicity towards HaCaT keratinocytes was also determined. Based on the results, the acetone–water extract from *V. myrtillus* was selected for further study. Using the Design of Experiments approach, chitosan-based hydrogels with bilberry fruit extract were prepared. The content of extract and chitosan were selected as independent factors. The activity of hydrogels depended on the extract content; however, the enzyme-inhibiting (anti-hyaluronidase and anti-tyrosinase) activity resulted from the presence of both the extract and chitosan. Increased concentration of chitosan in the hydrogel base led to increased viscosity of the hydrogel and, consequently, a slower release of active compounds. To get optimal hydrogel characteristics, 1% extract and 2.5% MMW chitosan were utilized. The research suggests the validity of using bilberry fruit extracts in topical preparations with anti-aging properties.

## 1. Introduction

Skin aging is a natural physiological process that depends on increasing intrinsic factors such as genetics, cell damage, and changes in the intercellular matrix. Repeated excessive exposures to UV solar radiation and other harmful environmental factors induce skin cell alterations similar to those observed during the aging process [1]. Thus, UV radiation absorbed by skin leads to an increase in the concentration of reactive oxygen species (ROS) in the tissues and, as a consequence, may cause lipid peroxidation, DNA damage, and modification of proteins and genes [2,3]. Moreover, the presence of ROS may stimulate the activity of some enzymes, including hyaluronidase, which destroys hyaluronic acid, and is one of the factors involved in maintaining the proper configuration of elastin and collagen fibers in the skin also ensuring proper skin hydration [4]. Therefore, a reduced amount of hyaluronic acid decreases skin elasticity and firmness [4]. Because of that, current strategies to decrease the skin aging process include the inhibition of enzymes that can destroy the structural integrity of the skin, e.g., hyaluronidase [5]. It has also been shown that UV radiation-induced ROS cause the secretion of proinflammatory cytokines in human dermal fibroblasts [6]. This results in local damage [7], which can lead to skin illnesses and accelerate the skin aging process [8]. Moreover, the high level of ROS in the skin tissue increases the melanogenesis process. This results from the impact of ROS on keratinocytes adjacent to melanocytes, and as a consequence, the action of melanogenic factors—tyrosinase (the enzyme responsible for the biosynthesis of the skin pigment melanin) and tyrosine-related proteins—is augmented [9]. Because melanin is one of the hallmarks of skin aging [10], the inhibition of tyrosinase decreases age-evoked hyperpigmentation. The anti-pigmentation effect is desirable in anti-aging cosmetic products. 

Scientific research has shown that plant extracts rich in polyphenols can reduce the level of free radicals in the skin and inhibit hyaluronidase and tyrosinase activity [11]. This antioxidant and anti-enzymatic potential of plant extracts is eagerly used in the modern cosmetic and pharmaceutical industry to prepare plant topical products that delay skin aging. *Vaccinium myrtillus* L. (bilberry) and *Vaccinium corymbosum* L. (blueberry) are commonly known blueberry species from the *Ericaceae* family. They are rich in polyphenols flavonoids, including anthocyanins, and others phenolic compounds such as phenolic acids (e.g., chlorogenic acid), proanthocyanidins, and stilbene derivatives [12,13,14]. The high health-promoting potential of both bilberry and blueberry fruits and fruit extracts has been described by many authors, and encompasses significant antioxidant activity [13]. Bilberry and blueberry fruits also have high anti-inflammatory potential confirmed among others by the ability of blueberry preparations to lower tumor necrosis factor-α, interleukin (IL)-6, and IL-1β expression [15]. Moreover, it was proven that blueberry consumption may decrease the loss of collagen—a key component of connective tissue [16,17]. Thus, the fruits of *V. myrtillus* and *V. corymbosum* are interesting materials in preparing plant products that slow down skin aging processes, especially those related to oxidative stress.

A naturally occurring cationic copolymer, chitosan is very interesting for hydrogel structures. Because of its hydrophilic character and its capacity to be broken down by human enzymes, this polymer is both biocompatible and biodegradable [18]. Chitosan is a natural wound-dressing substance because of its antibacterial, antioxidant, and immunomodulatory properties, which help stop wounds from becoming infected and encourage healing through the regeneration of soft tissues [19]. Moreover, due to its anti-inflammatory and antioxidant properties, as well as the ability to absorb UV rays, chitosan is used as an additive to anti-aging products [20].

The main goal of our study was to prepare, for the first time, chitosan-based hydrogels enriched in bilberry or blueberry dry fruit extracts characterized by high biological potential. Thus, first, we tested different fruit extracts from *V. myrtillus* and *V. corymbosum* to select the extract with high polyphenols content and the most beneficial biological properties. Then, we optimized the process of designing hydrogels by adding the selected extract, assessing its biological potential, and characterizing its pharmaceutical properties.

## 2. Materials and Methods

### 2.1. Plant Material

The plant material (dried *V. corymbosum* fruits and *V. myrtillus* fruits) were purchased from BadaPak, Poland (*V. corymbosum*), and from Kawon, Poland (*V. myrtillus*).

### 2.2. Chemical Reagents

Aluminum chloride hexahydrate, sodium carbonate, sodium hydroxide, hydrochloric acid, acetone, methanol, were purchased from Avantor Performance Materials Poland S.A. (Gliwice, Poland). The Folin-Ciocalteu′s phenol reagent was from Merck (Darmstadt, Germany). DMEM and antibiotics were purchased from Biowest (Nuaillé, France), FBS was obtained from EURx (Gdansk, Poland). Chitosan medium molecular weight, CS MMW (200–800 cP, 1 wt. % in 1% acetic acid) was supplied by Sigma-Aldrich (Poznan, Poland) as well as all the other chemicals (2,2-Diphenyl-1-picrylhydrazyl (DPPH), sodium chloride, bovine serum, hexadecyltrimethylammonium bromide (CTAB), hyaluronic acid (HA), phosphate buffer), tyrosinase, 3,4-Dihydroxy-L-phenylalanine (L-DOPA), quercetin, gallic acid.

### 2.3. Optimization of Extraction Process of Vaccinium myrtillus and Vaccinium corymbosum Fruits Regarding Its Biological Activity

The first part of the study was dedicated to choosing the most promising extracts from one of the *Vaccinium* species’ dry fruits. For this purpose, the extracts were prepared using four solvents in the first step. Subsequently, phytochemical characterization (the total content of polyphenols, flavonoids, and anthocyanins) was performed using spectrophotometric analysis. The screening of the biological potential of the obtained extracts was effectuated by in vitro methods. Antioxidant properties were determined using DPPH analysis; the ability to inhibit hyaluronidase and tyrosinase was also tested. Finally, the cytotoxicity of the extracts towards human keratinocytes (HaCaT) was determined using the MTT method.

#### 2.3.1. Extracts Preparation

To obtain extracts with different phytochemical and biological characteristics, 5.0 g of finely ground dry fruits of *V. myrtillus* or *V. corymbosum*, was extracted using an ultrasonic bath (40 °C). The extraction process was repeated four times, each for 20 min, using fresh portions (50 mL) of an appropriate solvent (methanol, water, methanol–water 1:1 *v*/*v*, or acetone–water 1:1 *v*/*v*). The obtained extracts were filtered and concentrated using a rotary evaporator to a volume of 50 mL (0.1 g fruit dry weigh/mL) and kept until further studies at −20 °C.

#### 2.3.2. Total Polyphenols Content, Total Flavonoids Content, Total Anthocyanins Content Evaluation, and Content of Active Compound

Total polyphenolic content (TPC) was examined using the Folin-Ciocalteau method [21]. The gallic acid calibration curve was used to calculate the content of polyphenolic compounds, and the obtained results were presented as mg gallic acid equivalent/g of fruit dry weight (mg GAE/g of FDW). The average from *n* = 6 measurements was presented. 

Total flavonoids content (TFC) was determined using the AlCl_3_ methods [21]. The quercetin calibration curve was used to calculate the content of flavonoids, and the obtained results were presented as mg quercetin equivalent/g of fruit dry weight (mg QE/g of FDW). The average from *n* = 6 measurements was presented.

For the evaluation of total anthocyanins content (TAC) in *V. myrtillus* and *V. corymbosum* dry fruits, the spectrophotometric method from the Polish Pharmacopeia was performed which takes into account the European Pharmacopoeia 9.0 regulations [22]. The extract was filtered and the dilution in 0.1% *v*/*v* HCl in methanol was used to measure absorbance. The results were presented as a percentage of cyanidin 3-glucoside. The average from *n* = 2 measurements was presented. 

The chlorogenic acid content in prepared extracts were determined with the previously described HPLC method [23] at a detection wavelength of 325 nm (Figure S1, Supplementary Material). The method was validated for chlorogenic acid, and the validation parameters are presented in Table S1 (Supplementary Material).

#### 2.3.3. Biological Activity Evaluation

Antioxidant activity was tested using methods assessing the ability of the extracts to scavenge free radicals (DPPH assay).

The DPPH analysis was performed using the protocol described previously by Studzińska-Sroka et al. [21]. The fluid extracts were tested and the concentration of the tested sample extracts was 12.5 mg/mL to 0.391 mg/mL (concentrations of the examined samples in the reaction mixture were 1.56 μg/mL to 0.05 μg/mL in the tested sample). The blanks and controls contained the solvent of the examined extracts in exchange for extract in the tested sample. The results were expressed as IC_50_ (mg DW/mL). The average from *n* = 2 measurements was presented. 

The enzyme inhibitory assays were performed in vitro using hyaluronidase and tyrosinase. The anti-hyaluronidase activity was measured using the procedure described by Studzińska-Sroka et al. [24]. The fluid extracts were tested and the concentration of the tested extract samples was 50 mg/mL (concentration of the examined samples in the reaction mixture was 5.0 mg/mL). The controls and blanks contained the solvent of the examined extracts instead of the sample. Absorbance was measured at 600 nm (Multiskan GO 1510, Thermo Fisher Scientific, Vantaa, Finland). The results were expressed as % of enzyme inhibition. The average from *n* = 5 measurements was presented. 

The anti-tyrosinase activity was evaluated using the protocol described previously [24] with some modifications. The fluid extracts were tested and the concentration of the tested extract sample was 10 mg/mL (concentration of the examined samples in the reaction mixture were 0.25 mg/mL). Therefore, the concentration of several reagents was adjusted (4 mM L-DOPA solutions was used), and incubation times during the experiment were 10 min and 25 min. The controls and blanks contained the solvent of the examined extracts instead of the sample. Absorbance was measured at 475 nm (Multiskan GO 1510, Thermo Fisher Scientific, Vantaa, Finland). The results were expressed as % of enzyme inhibition. The average from *n* = 3 measurements was presented. 

#### 2.3.4. Cell Viability Assay

The evaluation of the effect of the studied extracts was performed using the MTT assay, as previously described [25]. Briefly, HaCaT immortalized keratinocytes (Cell Lines Service, Eppelheim, Germany) were grown in DMEM supplemented with 10% FBS and 1% antibiotics solution. Cells were seeded into 96-well plates, and after 24 h pre-incubation, fresh medium containing the increasing concentrations of the studied extracts was added into wells, and cells were incubated for a subsequent 72 h. Next, wells were rinsed with warm PBS buffer, and fresh medium supplemented with 0.5% MTT salt was added into wells. Absorbance (570 nm, Infinite M200 plate reader, Tecan, Austria) was measured after 4 h incubation followed by dissolving formazan crystals using acidic isopropanol. Experiments were repeated four times with at least four technical replicates per assay. Impact on viability was calculated as percentage of results obtained for cells treated with the appropriate vehicle only.

### 2.4. Obtaining Hydrogels Containing Blueberry Fruit Extract

Using the Design of Experiments (DoE) approach, chitosan-based hydrogels were prepared with evaporated acetone–water (1:1 *v*/*v*) extracts from dried blueberry fruits. The content of extract and chitosan (medium molecular weight) were selected as independent factors. To select the optimal composition of hydrogel, a two-valued fractional plan was proposed (Statistica 13.3 software, TIBCO Software Inc., Palo Alto, CA, USA) and is presented in Table 1.

The parameters used to assess hydrogel properties were chosen: antioxidant activity (using DPPH scavenging assay), hyaluronidase inhibition, tyrosinase inhibition, dissolution profiles of standards from hydrogels, and their viscosity.

To prepare the hydrogels, water was weighed into the beaker, and the appropriate amount of extract was added and stirred for 5 min. Chitosan was then added and dissolved by adding 1% acetic acid. The hydrogel was stirred for 24 h before testing.

#### 2.4.1. Biological Activity Evaluation

Antioxidant, anti-hyaluronidase, and anti-tyrosinase activities were measured using the methods described above.

#### 2.4.2. Release of Active Compound

Vertical Franz cells (PermeGear, Inc., Hellertown, PA, USA) holding 5 mL of acceptor solution (phosphate buffer; pH = 5.5) were used for the hydrogel in vitro release tests. Membranes made of regenerated cellulose (^®^Nalo Cellulose, Kalle GmbH, Wiesbaden, Germany) with pore diameters of about 25 Å were installed in the cells. Prior to the experiment, the membranes were in the acceptor fluid at 37.0 ± 0.5 °C for a full day. Gel samples (1.0 mL) were put on the artificial membrane’s surface at the donor compartment and distributed uniformly. The cells in use have an effective diffusion area of 0.64 cm^2^. During the test, the temperature of the receptor fluid was maintained at 37.0 ± 0.5 °C and it was swirled at 400 rpm. At the designated intervals, 2.0 mL samples were removed from the acceptor compartment and quickly replaced with an equivalent volume of brand-new acceptor fluid. The chlorogenic acid concentrations in the collected samples were determined with the HPLC method described in Section 2.3.2. 

#### 2.4.3. Bioadhesive Properties Evaluation

A viscometric method was used to predict bioadhesive properties [26]. The viscometer AMETEK Brookfield DV2T (Middleborough, MA, USA) was used to measure the viscosity of the hydrogels that were created.

### 2.5. Statistical Analysis

The obtained data were expressed as the means ± SD. Statistical analysis was performed using a one-way analysis of variance (ANOVA), and statistical differences (using Duncan’s post hoc tests) with a significance threshold of p 0.05 were determined using Statistica 13.3 software (Statsoft, Krakow, Poland). Correlations were examined using principal component analysis (PCA) with PQStat Software version 1.8.4.142 and Statistica 13.3. 

## 3. Results and Discussion

### 3.1. Optimization of Extraction Process of Vaccinium myrtillus and Vaccinium corymbosum Fruits Regarding Its Biological Activity 

Polyphenols (including flavonoids or anthocyanins) determine the pleiotropic bioactivity of plant products. The most frequently described biological properties are antioxidant and anti-inflammatory potential. Moreover, their chemopreventive importance is also indicated [13]. Since ROS, generated both during the physiological aging process and under the influence of external factors (e.g., UV solar radiation), play an important role in the skin aging process [1], the polyphenol content may be one of the criteria for assessing the biological potential of natural raw materials.

Considering that some of the more important plant polyphenols are flavonoids and anthocyanins, we evaluated the TPC, TPF, and TAC. Our analyses prove that extracts prepared from both *Vaccinium* species contain compounds with a polyphenol structure, among which a certain pool consists of substances with a chemical structure of flavonoids and anthocyanins. Our results show that MeOH-H_2_O, H_2_O, and Ace-H_2_O extracts from *V. myrtillus* contain a higher polyphenol content than extracts from *V. corymbosum* (Table 2). Moreover, *V. myrtillus* extracts have a higher flavonoid and total anthocyanin content than *V. corymbosum* ones. Among all the tested extracts, the H_2_O ones usually had the lowest concentration of polyphenolic substances. To the best of our knowledge, no previous studies compare extracts prepared with dry fruits of *V. myrtillus* and *V. corymbosum*. However, it is worth noting that anthocyanin content results are consistent with earlier reports, which indicated 3–4 times higher anthocyanin contents in frozen bilberry than in blueberry fruits [14].

Further, the content of chlorogenic acid in four types of prepared *V. myrtillus* extracts was assessed (Table 3). The highest content was determined in the Acu-H_2_O extract.

**Figure 1:**
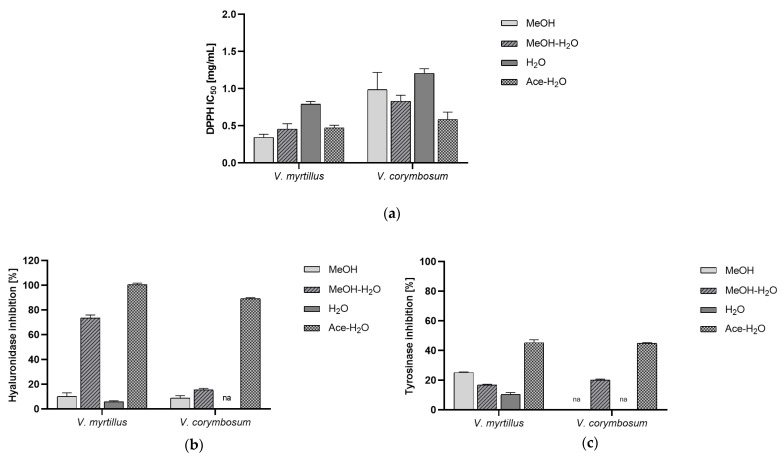
The biological activity of *Vaccinium myrtillus* and *Vaccinium corymbosum* extracts; the antioxidant potential (**a**), the anti-hyaluronidase potential (**b**), the anti-tyrosinase potential (**c**); na—not active.

Oxidative stress caused by excessive ROS generation contributes to the skin aging process. It disrupts the existing homeostasis of biological processes, leading to the peroxidation of the lipids of plasmatic membranes and organelles in cells [13]. All of these processes contribute to the loss of the primary properties of the skin. Thus, the ability to scavenge free radicals was measured to evaluate the antioxidant potential of *V. myrtillus* and *V. corymbosum* extracts. The results indicate that Ace-H_2_O extract from *V. myrtillus* and MeOH, MeOH-H_2_O as well, strongly scavenged free radicals in the DPPH test (Figure 1a). We showed that the antioxidant activity of *V. corymbosum* extracts, among which the Ace-H_2_O extract had the highest antioxidant activity, was lower than that of *V. myrtillus*. However, our results demonstrate the interesting antioxidant activity of *Vaccinium* dry fruits, which corroborate previous reports highlighting the powerful antioxidant effect of some *Vaccinium* species extracts evaluated with different antioxidant tests [27,28]. 

The proper hydration of skin protects against the appearance of wrinkles and the harmful effects of the environment. Hyaluronic acid determines proper hydration; hence, the higher its content in the intercellular matrix, the better the skin’s hydration. Moreover, the literature data indicate that low-chain fragments of hyaluronic acid correlate with tissue inflammation [29]. Since the presence of high-chain hyaluronic acid molecules in the skin determines the maintenance of the skin in good condition, inhibiting the breakdown processes of hyaluronic acid by blocking the activity of hyaluronidase has become the subject of research on the activity of blueberry and blueberry fruit extracts. We have proven that the extracts with the highest ability to inhibit hyaluronidase, regardless of the tested species, are the Ace-H_2_O extracts (Figure 1b). Thus, at the tested concentration (0.05 g/mL), they were characterized by very high hyaluronidase inhibitory activity (>90%). Methanol–water extracts also had a strong effect. On the other hand, water and methanol extracts from *V. corymbosum* did not inhibit the enzyme at all. Data on the hyaluronidase inhibitory properties of extracts from dried blueberries of the *Vaccinium* sp. are limited and concern species other than *V. myrtillus* and *V. corymbosum*. 

Tyrosinase is a key enzyme that controls the production of melanin in the skin. Because the aging process is related to higher activity of tyrosinase, substances which inhibit this enzyme can prevent aging pigmentation [30]. Therefore one of our goals was the evaluation of the inhibitory effect of *Vaccinium* spp. extracts on tyrosinase activity. The results (Figure 1c) have indicated that in the tested concentration, all of the *V. myrtillus* extracts blocked the activity of the enzyme. On the other hand, the *V. corymbosum* extracts’ activity was more varied and some of the extracts were inactive (MeOH and H_2_O). Moreover, a higher inhibition of tyrosinase was detected for the Ace-H_2_O extracts. Alhough, some of the *Vaccinium* spp. preparations are reported as tyrosinase inhibitors [31,32].

**Figure 2:**
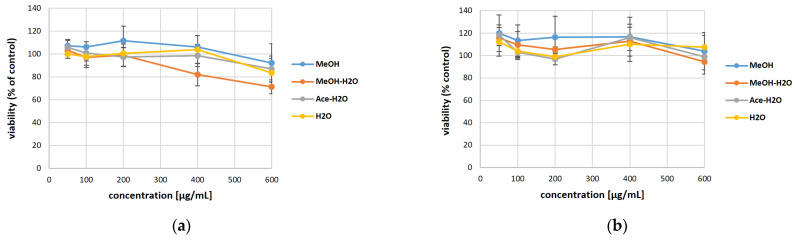
The effect of *Vaccinium myrtillus* (**a**) and *Vaccinium corymbosum* (**b**) extracts on the viability of HaCaT cells.

To assess the potentially cytotoxic effect of the extracts obtained from *V. myrtillus* and *V. corymbosum* on the skin cells, we performed the MTT test (Figure 2). The experiment was conducted in a model with the HaCaT cell line (normal human keratinocytes), which is currently most frequently used in skin research. Our results proved that in the concentrations used, the extracts from blueberry dry fruits are not cytotoxic (HaCaT viability > 90%). On the other side, MeOH, Ace-H_2_O, and H_2_O extracts from *V. myrtillus* show an HaCaT viability of 80–90%, which proves its very low cytotoxicity in a very high concentration (600 μg/mL). The obtained results suggest that these extracts have a high safety profile. This is consistent with the data of other authors who assessed the toxicity of blueberries in relation to normal HaCaT cell lines as low or not disturbing cell viability [33,34]. The low cytotoxicity we detected, especially for *V. myrtillus* (revealed only at very high concentrations), may result from the important content of polyphenolic compounds (including flavonoids and anthocyanins) in the tested extracts. The content of Anthocyanins in bilberry extracts is higher, which can contribute to stronger effects on cell viability. Indeed, the activity of berry juices could be attributed to the total anthocyanin content or to specific anthocyanin compounds [35]. Polyphenols are a group of substances with health-promoting significance, including high antioxidant potential [36], and a beneficial effect on the regulation of redox homeostasis in cells. However, the literature indicates that under certain conditions (e.g., high concentration), polyphenols may have a pro-oxidant effect [33], which may impair cell viability. A recent study has shown that bilberry extract may show pro-apoptotic activity through a redox-sensitive caspase 3 activation-related mechanism [37]. 

From the results obtained in this preliminary section, taken together, we conclude that the Ace-H_2_O *V. myrtillus* extract exerts a high antioxidant potential, and their inhibitory activity on hyaluronidase and tyrosinase is the highest in the tested concentration among all the tested extracts. Importantly, the content of chlorogenic acid is the highest in Ace-H_2_O. Chlorogenic acid is a compound with antioxidant and anti-inflammatory potential [38], as well as the ability to stimulate the upregulation of skin barrier genes [39]. It indicates that the Ace-H_2_O extract is an interesting material for our further studies. In addition, the *V. myrtillus* and *V. corymbosum* extracts are characterized by low cytotoxicity in the MTT assay. Considering all the obtained results, the Ace-H_2_O *V. myrtillus* extract was chosen for the subsequent studies. 

### 3.2. Obtaining Hydrogels Containing Blueberry Fruit Extract 

Based on the results of the cytotoxicity assessment of the extract, it was estimated that the maximum concentration of the extract in the hydrogel was 1%. This concentration does not cause any toxic effect.

The preparation of all hydrogels was carried out without any problems. All the samples were converted to gels in room temperature in <10 min. The aim of the study was to assess the influence of the composition of hydrogels on the biological and pharmaceutical properties of the systems. The assessment of the morphological structure was not in focus, but instead, the antioxidant, anti-hyaluronidase, and anti-tyrosinase properties were assessed first (Table 4).

The antioxidant activity of hydrogels results primarily from the presence of the extract and increases with an increase in its concentration in the hydrogel (Figure S2, Supplementary Material). As previously shown, the antioxidant activity of chitosan (CS) is negligible, which is due to insufficient H-atom donors [40]. On the other hand, the analysis of data on anti-hyaluronidase activity indicates that the activity of CS is much higher than that of the extract. It has been shown that the inhibition of hyaluronidase increases with an increase in the concentration of CS in the hydrogel and a decrease in the extract concentration (Figure S3, Supplementary Material). The process by which hyaluronidase’s activity is inhibited could be associated with the interaction of an increasing number of -NH_2_ groups of CS, perhaps resulting in modifications to the enzyme’s secondary structure [40]. Finally, anti-tyrosinase activity was assessed, which showed similar relationships as in the case of anti-hyaluronidase activity. Namely, with an increase in CS concentration, the activity of the hydrogel increases, and at the same time, it increases with a decrease in extract concentration (Figure S4, Supplementary Material). The literature data suggest that CS can inhibit tyrosinase activity by binding a copper ion in the active site of the enzyme and by binding to the active site of the enzyme, rather than changing the tertiary structure of tyrosinase [41,42].

**Figure 3:**
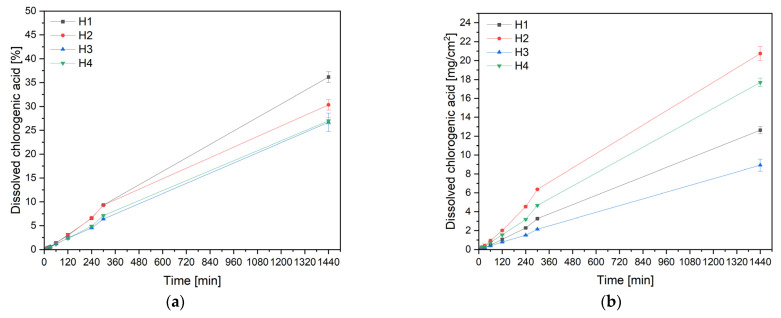
Release profiles of chlorogenic acid expressed in % (**a**) and expressed in mg/cm^2^ (**b**) for hydrogels H1–H4.

The next aspect was the assessment of the dissolution behavior of chlorogenic acid, as one of the main active compounds of the extract (Figure S4, Supplementary Material). So, chlorogenic acid’s release rate from hydrogels was estimated by in vitro release tests (Figure 3). After 24 h, the release was only 26–36% depending on the formulation. In this case, the cumulative amount of released chlorogenic acid should also be taken into account. The greatest amount of chlorogenic acid was released from formulation H2. Evidently, in this case, the most important factor influencing the amount of chlorogenic acid released was extract concentration (Figure S6, Supplementary Material). Moreover, in both cases, the release of the substance is influenced by the concentration of chitosan. The increase in CS concentration is correlated with the increase in hydrogel viscosity, which effectively slows down the release of chlorogenic acid (Figures S5 and S6, Supplementary Material). Comparing the similarity of the profiles, it was noticed that the profiles for H1 and H2, and H3 and H4 are similar, which shows again the strong influence of CS, because in the first two hydrogels, the CS concentration is 2%, and in the next ones, it is 3%, which significantly indicates the determination of the percentage of substance released by CS (Table S2, Supplementary Material).

The release profiles indicate that the drug release rate is independent of concentration and that, for each formulation, the process may be satisfactorily connected to and explained by the zero-order kinetics model (Table S3, Supplementary Material). This confirms the literature reports on the usefulness of chitosan for constructing systems for the controlled delivery of active substances [43,44].

One of the key factors influencing a system’s ability to be bioadhesive is its viscosity. So, the systems’ viscosity was examined (Table 5). Previous work assessed the impact of both molecular weight (MW) and the degree of deacetylation on the viscosity of the resulting systems, where it was shown that the viscosity increases with increasing MW. Therefore, only CS MMW, which shows optimal parameters, was used in this work. Of course, as the CS concentration increases, the lightness increases, which can be easily seen on the Pareto chart (Figure S7, Supplementary Material). The creation of mutually entangled chains between two contacting interfaces and charge interactions are the primary causes of chitosan hydrogels’ capacity for tissue adhesion [45]. Be aware that basic CSs may not adhere as well as modified ones, which are created by conjugating various adhesive agents to the CS backbone [46].

**Figure 4:**
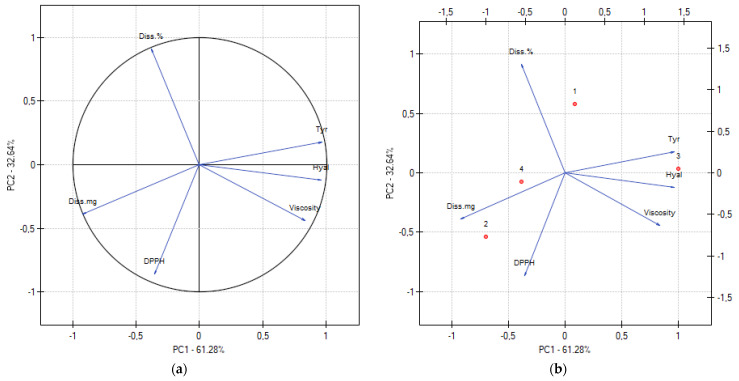
The relationship of hydrogels properties on the factorial plane formed by the first two principal components (**a**), and principal component analysis (PCA) showing the factor loading plot (**b**).

To evaluate the correlation of all experimental results, PCA analysis was performed (Figure 4; Table S4, Supplementary Material). A strong negative correlation was found between the viscosity of the hydrogels and the release of chlorogenic acid, which confirms the previously obtained results. Additionally, a correlation was demonstrated between the viscosity of anti-hyaluronidase and anti-tyrosinase activity, which indicates a weakening of the anti-aging effect with an increase in the viscosity of the system, mainly resulting from an increase in the concentration of chitosan.

**Figure 5:**
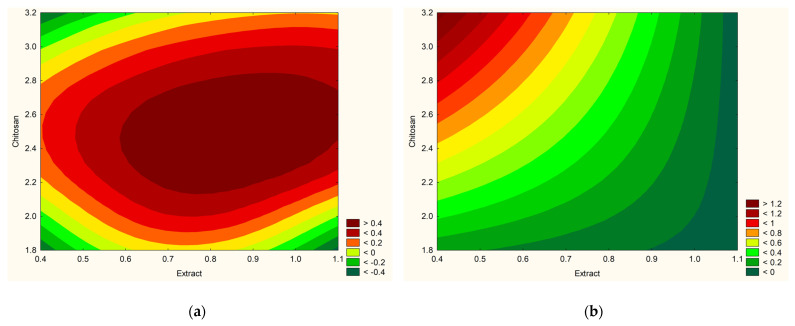
Prediction of the optimization model for obtaining hydrogels for effects with positive sign—antioxidant activity, dissolution and viscosity (**a**) and with negative sign—anti-hyaluronidase and anti-tyrosinase activities (**b**).

Using experimental data and statistical analysis, it was possible to estimate the model and determine the hydrogel’s optimal composition (Figure 5). To get optimal hydrogel characteristics, 1% extract and 2.5% MMW chitosan were utilized.

## 4. Conclusions

In conclusion, our research confirms that *V. myrtillus* and *V. corymbosum* dry fruit extracts possess important phytochemical and biological potential that can differ respectably from the species and solvents used for extraction. This diversity allowed us to select the most valuable extract for further research. We also proved that the DoE approach can be successfully used to determine the optimal parameters to prepare hydrogels with interesting biological and physicochemical properties. The parameters of hydrogel, along with the proven antioxidant, anti-hyaluronidase, and anti-tyrosinase properties of prepared hydrogels, suggest the anti-aging potential of prepared *V. myrtillus* extracts in a topical preparation.

## Figures and Tables

**Table 1:** Factorial hydrogel preparation experiment plan.

	Extract [%]	Chitosan MMW [%]
H1	0.5	2.0
H2	1.0	2.0
H3	0.5	3.0
H4	1.0	3.0

**Table 2:** Total polyphenols content (TPC) and total flavonoids content (TFC) in *Vaccinium myrtillus* and *Vaccinium corymbosum* extracts.

Extracts	*V. myrtillus*	*V. corymbosum*	*V. myrtillus*	*V. corymbosum*	*V. myrtillus*	*V. corymbosum*
	mg GAE/g DW		mg QE/g DW		% Cyanidin 3-Glucoside	
MeOH	17.62 ± 0.78 ^c^	22.3 ± 0.91 ^b^	1.83 ± 0.23 ^a^	0.989 ± 0.02 ^a^	0.50 ± 0.03 ^c^	0.24 ± 0.01 ^a^
MeOH-H_2_O	35.59 ± 1.30 ^a^	18.43 ± 0.90 ^c^	1.43 ± 0.09 ^b^	0.488 ± 0.01 ^b^	0.70 ± 0.02 ^a^	0.27 ± 0.02 ^a^
H_2_O	18.57 ± 0.80 ^c^	17.79 ± 0.43 ^c^	0.73 ± 0.17 ^d^	0.258 ± 0.01 ^d^	0.34 ± 0.03 ^d^	0.26 ± 0.02 ^a^
Ace-H_2_O	28.24 ± 1.16 ^b^	28.17 ± 1.03 ^a^	1.16 ± 0.04 ^c^	0.321 ± 0.02 ^c^	0.51 ± 0.02 ^b^	0.24 ± 0.03 ^a^

Mean values within a column with the same letter are not significantly different at *p* < 0.05 using Duncan’s test. The first letter of the alphabet is used for the highest values, the next for statistically significant decreasing values; mg GAE/g DW—mg gallic acid equivalent/g of fruit dry weight; mg QE/g DW—mg quercetin equivalent/g of fruit dry weight.

**Table 3:** Chlorogenic acid content in *V. myrtillus* extracts.

Extracts	*V. myrtillus*
	µg Chlorogenic Acid/g Plant Material
MeOH	103.28 ± 2.10 ^d^
MeOH-H_2_O	152.10 ± 1.15 ^b^
H_2_O	108.26 ± 2.11 ^c^
Ace-H_2_O	175.67 ± 1.18 ^a^

Mean values within a column with the different letter are significantly different at *p* < 0.05 using Duncan’s test. The first letter of the alphabet is used for the highest values, the next for statistically significant decreasing values.

**Table 4:** Biological activities of hydrogels.

	Antioxidant Activity	Anti-Hyaluronidase Activity	Anti-Tyrosinase Activity
	Hydrogel activity [%]	IC_50_ [mg hydrogel]	IC_50_ [mg hydrogel]
H1	20.49 ± 3.36	32.11 ± 0.43	71.74 ± 9.30
H2	26.87 ± 7.54	16.05 ± 0.39	62.97 ± 7.03
H3	23.79 ± 5.06	116.26 ± 26.65	82.62 ± 3.88
H4	24.14 ± 3.89	25.89 ± 1.76	65.64 ± 13.59
CS	n/d	18.06 ± 2.95	48.52 ± 6.25
	IC_50_ [mg/mL]	IC_50_ [mg/mL]	IC_50_ [mg/mL]
Extract	1.01 ± 0.20	14.21 ± 1.43	2.21 ± 0.09
Chlorogenic acid	0.12 ± 0.01	>50.00 *	3.89 ± 0.69

* activity for concentration 50.00 mg/mL at level 14.30 ± 1.10%; CS—chitosan.

**Table 5:** Viscosity of hydrogels H1–H4.

	Viscosity [mPa s]
H1	128.50 ± 0.71
H2	87.50 ± 0.71
H3	501.50 ± 21.92
H4	379.00 ± 56.57
CS 2%	2187.50 ± 60.10
CS 3%	5681.00 ± 9.90

## Data Availability

The data supporting the reported results can be found in: Department of Pharmacognosy and Biomaterials, Poznan University of Medical Sciences; Department of Pharmaceutical Biochemistry, Poznan University of Medical Sciences.

## Supplementary Materials

The following supporting information can be downloaded at: https://www.mdpi.com/article/10.3390/antiox13010105/s1, Figure S1: Chromatogram of acetone–water extract at concentration 0.1 g/mL; Table S1. Validation parameters of HPLC method; Figure S2: Statistical analysis for antioxidant activity: (a) Pareto plot of standardized effects; (b) Response surface plots presenting the dependence of extract concentration and chitosan concentration on hydrogel antioxidant activity; Figure S3: Statistical analysis for anti-inflammatory activity: (a) Pareto plot of standardized effects; (b) Response surface plots presenting the dependence of extract concentration and chitosan concentration on hydrogel anti-hyaluronidase activity; Figure S4: Statistical analysis for anti-tyrosinase activity: (a) Pareto plot of standardized effects; (b) Response surface plots presenting the dependence of extract concentration and chitosan concentration on hydrogel anti-tyrosinase activity; Figure S5: Statistical analysis for release studies: (a) Pareto plot of standardized effects for chlorogenic acid release (in %); (b) Response surface plots presenting the dependence of extract concentration and chitosan concentration on the chlorogenic acid release from hydrogels (in %); Figure S6: Statistical analysis for release studies: (a) Pareto plot of standardized effects for chlorogenic acid release (in mg/cm^2^); (b) Response surface plots presenting the dependence of extract concentration and chitosan concentration on the chlorogenic acid release from hydrogels (in mg/cm^2^); Table S2: Comparison of chlorogenic acid release profiles (expressed in %) from H1–H4 hydrogels using factors *f*_1_ and *f*_2_; Table S3: Parameters of mathematical models fitted to the release profiles (expressed in mg/cm^2^) of formulations H1–H4; Figure S7: Statistical analysis for hydrogels’ viscosity: (a) Pareto plot of standardized effects; (b) Response surface plots presenting the dependence of extract concentration and chitosan concentration on hydrogels’ viscosity; Table S4: Correlation matrix.
