# Supplementary material for: Anti-Aging Properties of Chitosan-Based Hydrogels Rich in Bilberry Fruit Extract
Source: Antioxidants (Basel). 2024 Jan 15;13(1):105. doi: 10.3390/antiox13010105 (PMC10812676; doi:10.3390/antiox13010105)
Supplement: Supplementary file 1 [file antioxidants-13-00105-s001.zip › antioxidants-2777266-supplementary.pdf]

antioxidants

MDPI

Anti-Aging Properties of Chitosan-Based Hydrogels Rich in Bilberry Fruit Extract

Elżbieta Studzińska-Sroka [1], Magdalena Paczkowska-Walendowska [1,*], Cansu Erdem [2], Jarosław Paluszczak [3], Robert Kleszcz [3], Marta Hoszman-Kulisz [1] and Judyta Cielecka-Piontek [1]

[1] Department of Pharmacognosy and Biomaterials, Poznan University of Medical Sciences, Rokietnicka 3 Str, 60-806 Poznań, Poland; elastudzinska@ump.edu.pl (E.S.-S.); martahoszman@wp.pl (M.H.-K.); jpiontek@ump.edu.pl (J.C.-P.)
[2] Department Pharmaceutical Chemistry, Ege Üniversitesi, 35040 İzmir, Turkey; cansueerdem@gmail.com
[3] Department of Pharmaceutical Biochemistry, Poznan University of Medical Sciences, Rokietnicka 3 Str, 60-806 Poznań, Poland; paluszcz@ump.edu.pl (J.P.); kleszcz@ump.edu.pl (R.K.)
* Correspondence: mpaczkowska@ump.edu.pl

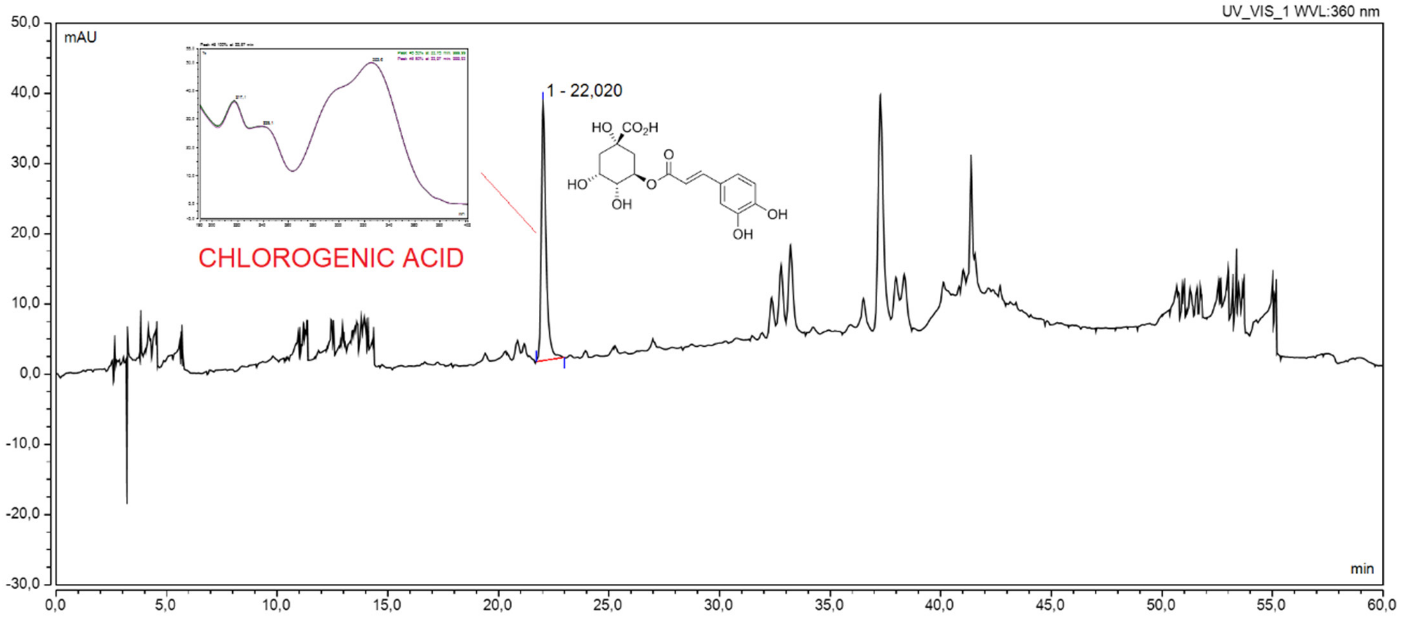

Figure S1. Chromatogram of aceton-water extract at concentration 0.1 g/ml.

Table S1. Validation parameters of HPLC method

Parameter	**Chlorogenic acid**
Linearity: y = ax + b	
a ± S_a	0.5068 ± 0.0054
b ± S_b	insignificant (α=0.05)
Correlation coefficient (r)	0.9998
Range of linearity [µg/mL]	21.2–212.0
Intra-day precision, RSD (<5% required) = repeatability	
The lowest	2.46
The middle	0.04
The lowest	0.13
Limit of detection (LOD) [µg/mL]	5.21
Limit of quantification (LOQ) [µg/mL]	15.79

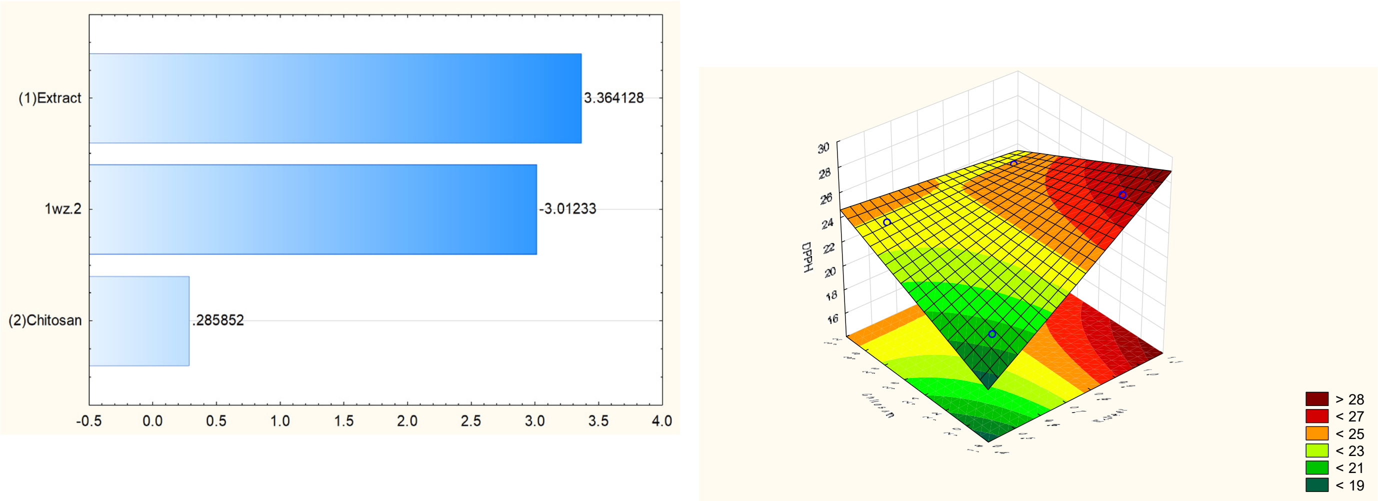

(a) (b)

Figure S2. Statistical analysis for antioxidant activity: (a) Pareto plot of standardized effects; (b) Response surface plots presenting the dependence of extract concentration and chitosan concentration on hydrogel antioxidant activity.

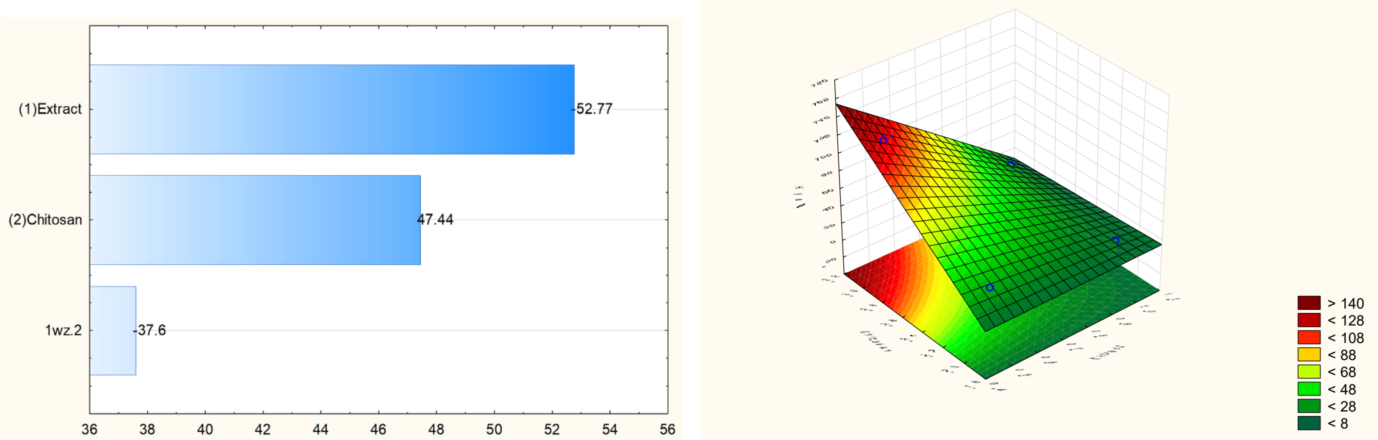

(a) (b)

Figure S3. Statistical analysis for anti-inflammatory activity: (a) Pareto plot of standardized effects; (b) Response surface plots presenting the dependence of extract concentration and chitosan concentration on hydrogel anti-hyaluronidase activity.

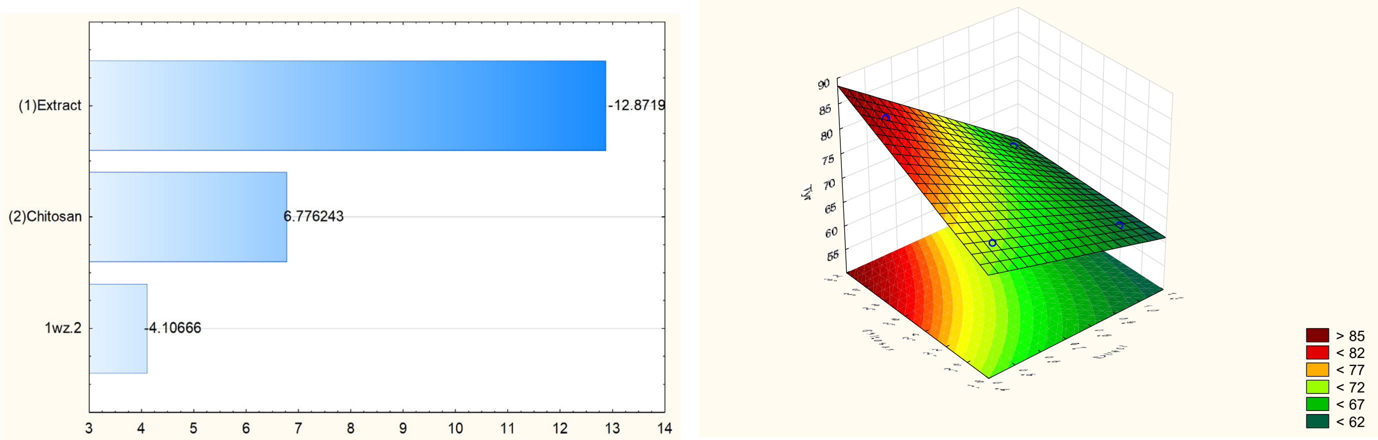

(a) (b)

Figure S4. Statistical analysis for anti-tyrosinase activity: (a) Pareto plot of standardized effects; (b) Response surface plots presenting the dependence of extract concentration and chitosan concentration on hydrogel anti-tyrosinase activity.

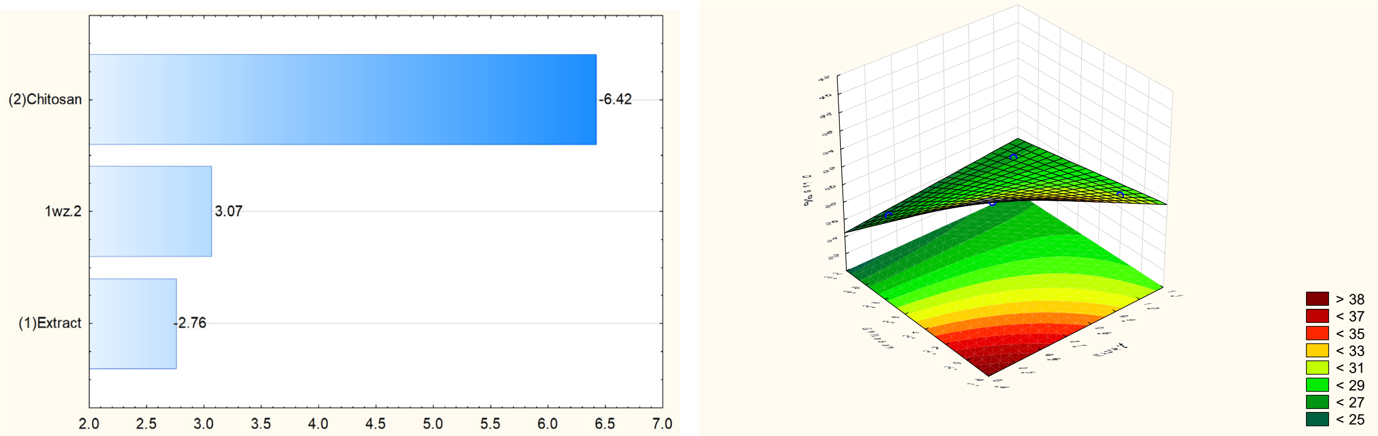

(a) (b)

Figure S5. Statistical analysis for release studies: (a) Pareto plot of standardized effects for chlorogenic acid release (in %); (b) Response surface plots presenting the dependence of extract concentration and chitosan concentration on the chlorogenic acid release from hydrogels (in %).

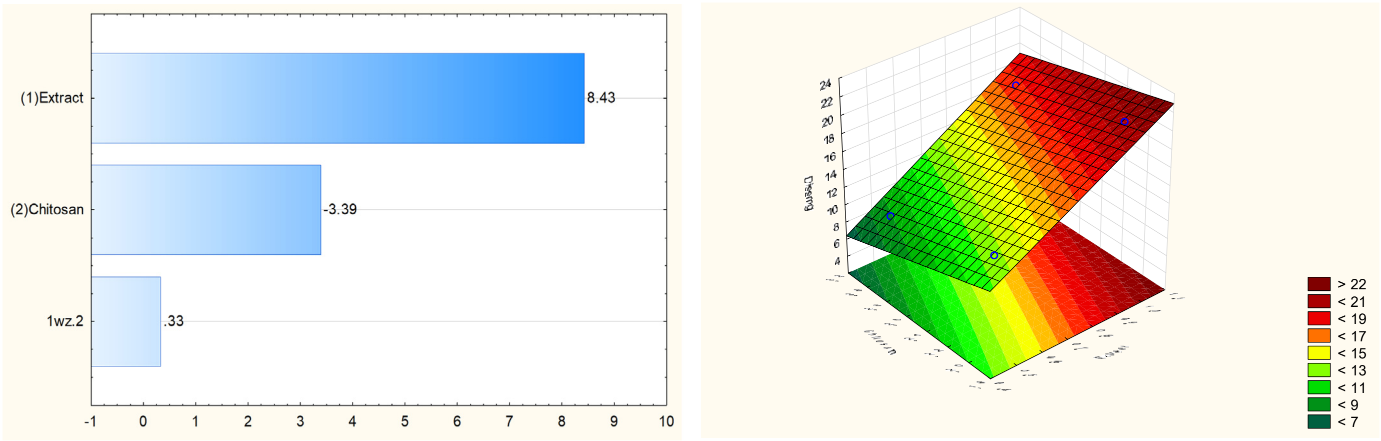

(a) (b)

Figure S6. Statistical analysis for release studies: (a) Pareto plot of standardized effects for chlorogenic acid release (in mg/cm^2); (b) Response surface plots presenting the dependence of extract concentration and chitosan concentration on the chlorogenic acid release from hydrogels (in mg/cm^2).

Table S2. Comparison of chlorogenic acid release profiles (expressed in %) from H1-H4 hydrogels using factors f_1 and f_2

	H1	H2	H3	H4
H1		f_1=11.00<br>f_2=82.45	f_1=27.11<br>f_2=63.07	f_1=24.74<br>f_2=64.96
H2	f_1=11.00<br>f_2=82.45		f_1=18.54<br>f_2=72.66	f_1=15.99<br>f_2=75.58
H3	f_1=27.11<br>f_2=63.07	f_1=18.54<br>f_2=72.66		f_1=3.81<br>f_2=97.88
H4	f_1=24.74<br>f_2=64.96	f_1=15.99<br>f_2=75.58	f_1=3.81<br>f_2=97.88	

similar profiles marked in red

Table S3. Parameters of mathematical models fitted to the release profiles (expressed in mg/cm^2) of formulations H1-H4

Formulation No.	Mathematical model							
	Zero-order kinetic		First-order kinetic		Higuchi kinetic		Korsmeyer-Peppas kinetic	
	K	R^2	K	R^2	K	R^2	R^2	n
H1	**0.53**	**0.99**	0.15	0.57	1.09	0.54	0.44	0.37
H2	**0.86**	**0.99**	0.15	0.64	1.87	0.59	0.67	0.42
H3	**0.37**	**0.99**	0.14	0.62	0.76	0.53	0.38	0.30
H4	**0.74**	**0.99**	0.15	0.67	1.53	0.55	0.59	0.39

the most fitting mathematical model is shown in bold

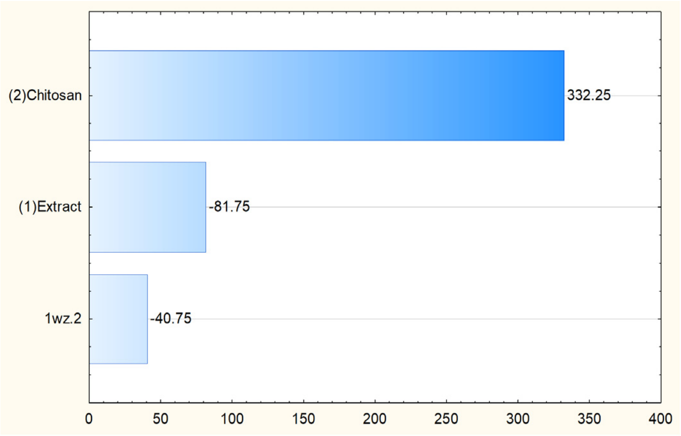

(a)

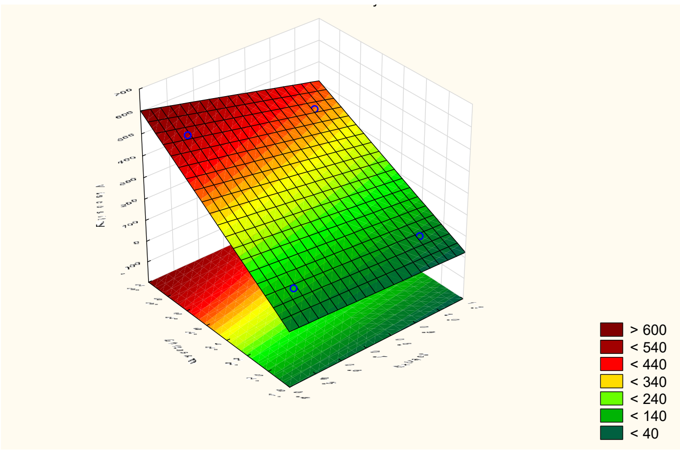

(b)

Figure S7. Statistical analysis for hydrogels' viscosity: (a) Pareto plot of standardized effects; (b) Response surface plots presenting the dependence of extract concentration and chitosan concentration on hydrogels' viscosity.

Table S4. Correlation matrix

	**DPPH**	**Hyal**	**Tyr**	**Diss.%**	**Diss.mg**	**Viscosity**
**DPPH**	1.0000	-0.1479	-0.4240	-0.5894	0.6419	-0.0369
**Hyal**	-0.1479	1.0000	0.9544	-0.4396	-0.8510	0.7779
**Tyr**	-0.4240	0.9544	1.0000	-0.1782	-0.9665	0.6663
**Diss.%**	-0.5894	-0.4396	-0.1782	1.0000	-0.0073	-0.7729
**Diss.mg**	0.6419	-0.8510	-0.9665	-0.0073	1.0000	-0.5837
**Viscosity**	-0.0369	0.7779	0.6663	-0.7729	-0.5837	1.0000

statistically significant relationships are marked in red
